# Applications of Left Atrial Strain in Cardiovascular Disease: A Comprehensive Review of Recent Evidence

**DOI:** 10.31083/RCM48668

**Published:** 2026-08-19

**Authors:** Jiajun Chen, Guoxing Ling, Chen Fang, Zimin Wu, Shigao Ye, Zuyuan Huang, Cheng Luo, Baoshi Zheng

**Affiliations:** ^1^Department of Cardiovascular Surgery, The First Affiliated Hospital of Guangxi Medical University, 530021 Nanning, Guangxi, China

**Keywords:** left atrial function, echocardiography, myocardial strain, heart failure, atrial fibrillation, hypertension, heart valve diseases

## Abstract

Although left atrial (LA) function is of major clinical importance in cardiovascular diseases, traditional indicators such as LA volume struggle to promptly reflect functional changes. In recent years, the emerging technique of left atrial strain (LAS) has garnered widespread attention. As a quantitative metric based on speckle tracking echocardiography, LAS can detect subtle functional abnormalities in the LA myocardium at an early stage and independently assess atrial reservoir, conduit, and contraction functions beyond volume changes. Numerous recent studies have demonstrated the significant clinical utility of LAS across multiple cardiovascular conditions, including heart failure, atrial fibrillation, hypertension, valvular heart disease, and coronary artery disease. Furthermore, LAS exhibits prognostic value in cardiomyopathies and cancer therapy–related cardiotoxicity. This review summarizes recent advances in LAS across various cardiovascular diseases, objectively evaluates its clinical application prospects and limitations, and outlines future research directions.

## 1. Introduction

The left atrium (LA) is not merely a passive conduit but an active chamber involved throughout the cardiac cycle. Its physiological function can be summarized as three phases. The conduit phase occurs during early to mid-diastole, where the LA passively transmits blood flow to the left ventricle (LV), jointly regulated by the atrioventricular pressure gradient and LV diastolic compliance. The booster pump phase occurs in late diastole, where active contraction of sinus rhythm-driven atrial muscle contributes approximately 15–30% of LV filling volume. This becomes particularly critical when the LV filling pressure increases or diastolic function is impaired [1,2]. LA dysfunction leads to reduced cardiac output and hemodynamic instability, and is increasingly recognized as an independent pathological phenotype termed atrial failure [3]. Traditional methods for assessing LA function include the measurement of LA volume and pressure. However, these indicators often reflect functional impairment only after structural changes occur, thus limiting their sensitivity [4,5].

Left atrial strain (LAS) is a myocardial deformation parameter of the LA obtained using two-dimensional speckle tracking imaging in cardiac ultrasound. Compared to traditional volumetric indices, LAS can independently quantify the degree of deformation in LA myocardial fibers earlier and with greater sensitivity, irrespective of chamber size [5,6,7]. During early ventricular diastole, blood flows from the LA into the LV, thereby decreasing strain during the conduit phase. Strain reaches a negative peak during active atrial contraction at the end of the cardiac cycle (left atrial contraction strain, LASct), reflecting LA pump function [8]. In normal adults, LASr typically exceeds 30–40%, while conduit and contraction strains are relatively lower. Normal reference ranges have been proposed in multiple studies and consensus documents [9,10]. Given its cost-effectiveness, accessibility, and reproducibility in ultrasound acquisition, LAS has become one of the most important noninvasive parameters for evaluating LA function [11]. An early overview by Cameli et al. [12] synthesized the emerging evidence on LAS, highlighting its potential to confer incremental diagnostic and prognostic value beyond conventional echocardiographic parameters, including LA volume. This review was instrumental in framing LAS as a sensitive marker of atrial dysfunction across a broad spectrum of cardiovascular conditions. This seminal work laid the foundation for subsequent studies to explore the role of LAS as a sensitive marker of atrial dysfunction across a broad spectrum of cardiac conditions. The definitions and clinical interpretation of LAS parameters are shown in Table 1.

**Table 1.** **Definition and clinical interpretation of LAS parameters**.

Parameter	Phase of LA function	Definition	Primary physiological correlate	Common clinical interpretation
LASr	Reservoir phase	Peak positive LA longitudinal strain during ventricular systole	LA compliance and reservoir function	Reduced LASr suggests impaired LA compliance and elevated LV filling pressure
LAScd	Conduit phase	Difference between LASr and LASct	Passive LA emptying during early diastole	Reduced LAScd reflects impaired LV relaxation and diastolic dysfunction
LASct	Contractile phase	Strain during active atrial contraction	Atrial booster pump function	Reduced LASct indicates impaired atrial contractility
LASRr	Reservoir strain rate	Peak positive strain rate during reservoir phase	Rate of LA expansion	Sensitive to acute changes in LA pressure
LASRcd	Conduit strain rate	Early diastolic negative strain rate	Passive LA emptying velocity	Altered in early diastolic dysfunction
LASRct	Contraction strain rate	Late diastolic negative strain rate	Atrial contraction velocity	Reflects atrial systolic performance

LAS, left atrial strain; LA, left atrial; LV, left ventricle; LASr, left atrial reservoir strain; LAScd, left atrial conduit strain; LASct, left atrial contraction strain; LASRr, left atrial reservoir strain rate; LASRcd, left atrial conduit strain rate; LASRct, left atrial contraction strain rate.

In recent years, numerous studies have documented the clinical significance of LAS across various cardiovascular diseases [13]. A decrease in LAS often precedes structural changes such as LA enlargement, indicating subclinical cardiac dysfunction. Importantly, LAS levels correlate closely with the risk of cardiovascular events, making it valuable for patient risk stratification and assessment of prognosis. This review focuses on the major research achievements in recent years, detailing the application of LAS in heart failure (HF), atrial fibrillation (AF), hypertension, coronary heart disease (CHD), cancer therapy-related cardiotoxicity, valvular heart disease, and cardiomyopathy. We also discuss the clinical translational value of LAS and remaining challenges.

## 2. Measurement and Normal Values of Left Atrial Strain

**Measurement Method**: LAS is primarily obtained using two-dimensional speckle tracking echocardiography (2D-STE) via transthoracic echocardiography. During this procedure, the apical four-chamber (A4C) and two-chamber (A2C) views are used to focus on the LA. Tracking the motion of the LA endocardial border yields the LA longitudinal strain-time curve [14]. It is important to note that the image frame rate should be maintained at 60–80 frames per second in order to ensure accurate strain analysis. A lower frame rate may underestimate strain changes during rapid motion phases. For patients with arrhythmias (such as AF), it is recommended to average the strain values across multiple cardiac cycles to enhance accuracy [15].

**Normal Reference Range**: In healthy individuals, LAS exhibits characteristic variations across three cardiac phases: the reservoir phase strain (LASr) is maximal, with normal values of approximately 35–45%; the conduit phase strain (LAScd) ranges from 10–25%; while the contractile phase strain (LASct) is negative, with absolute values of approximately 10–20%. Multiple studies and expert consensus have established these reference ranges for the various LAS parameters; LASr holds the greatest clinical value as it reflects the maximum expansion capacity of the LA [10]. It is important to emphasize that LAS is influenced by factors such as age, heart rate, and hemodynamic status. The interpretation of measurement results should therefore be contextualized within the patient’s specific clinical circumstances [16].

## 3. Application of Left Atrial Strain in Heart Failure

### 3.1 Application of Left Atrial Strain in Heart Failure With Preserved Ejection Fraction

HF with preserved ejection fraction (HFpEF) is defined by clinical symptoms and signs consistent with HF, a left ventricular ejection fraction (LVEF) ≥50%, and objective evidence supporting diastolic dysfunction or elevated filling pressures [17]. The pathophysiology of HFpEF is primarily driven by LV diastolic dysfunction, leading to elevated LV filling pressures and subsequent LA pressure overload and structural/functional remodeling [18].

LAS provides a valuable tool for assessing LA function in HFpEF patients. Typically, HFpEF is associated with mild to moderate reductions in LASr and markedly decreased LAScd (indicating diminished LA compliance). Systolic strain may remain relatively preserved in the early stages but declines in later disease progression. Several clinical studies support the diagnostic and prognostic value of LAS in HFpEF. For instance, Ye et al. [19] reported that LASr can distinguish HFpEF patients from healthy controls, thereby serving as a robust indicator for assessing diastolic function (with a cutoff of approximately 23% for identifying diastolic dysfunction). A recent machine learning analysis further integrated LAS into HFpEF stratification and prognostic assessment: Carluccio et al. [20] performed machine learning clustering on 864 HF patients, identifying three distinct diastolic function states by combining LASr with conventional diastolic parameters. This LASr-based classification demonstrated superior correlation with clinical outcomes (rehospitalization/mortality) compared to traditional guideline classifications [20]. Recent ML studies have begun to incorporate strain-derived features (including atrial strain in selected cohorts) to refine diastolic function stratification and risk profiling in HF [21]. In an independent validation cohort, the classification incorporating LASr also showed superior risk prediction capability, demonstrating that integration of LAS improves diastolic function stratification and prognostic assessment in HF patients, including those with HFpEF [20].

Beyond diagnostic stratification, LAS also predicts clinical outcomes in HFpEF. Studies have shown that reduced LASr in HFpEF patients correlates with increased risks of HF hospitalization and mortality [22]. Reported LASr cut-off values in HFpEF vary across studies, generally ranging between 20% and 25%. Such variability likely reflects differences in patient characteristics and strain acquisition platforms. Therefore, LAS values should be interpreted cautiously, and fixed prognostic thresholds cannot yet be universally applied. However, LAS has not been officially included in the diagnostic criteria for HFpEF, and more prospective studies are needed in the future to verify its incremental value and determine the optimal threshold.

### 3.2 Application of Left Atrial Strain in Heart Failure With Mildly Reduced Ejection Fraction

HF with mildly reduced ejection fraction (HFmrEF, LVEF 41–49%) occupies an intermediate position between heart failure with reduced ejection fraction (HFrEF) and HFpEF, exhibiting clinical characteristics and prognosis that are a compromise between the two [23]. Although research on LAS in HFmrEF is relatively scarce, its trend is hypothesized to lie between HFrEF and HFpEF. Moderate diastolic dysfunction and moderately elevated filling pressures are commonly observed in HFmrEF patients and may contribute to some degree of LA remodeling and functional decline [24,25].

Some studies suggest the LAS parameters in HFmrEF patients may already be abnormal, but to a lesser extent than in HFrEF. For example, Lundberg et al. [26] reported that the median LASr in the HFmrEF group fell between that of the HFrEF and HFpEF groups and reflected changes in LA pressure. HFmrEF patients retain some compensatory capacity before ejection fraction decline, potentially resulting in relatively lower systolic strain compared to HFpEF, while the decrease in reservoir strain may be less pronounced than in HFrEF. Notably, significant LAS impairment in HFmrEF patients suggests a pathophysiology closer to HFrEF, potentially indicating a worsened prognosis [27]. Therefore, monitoring of changes in LAS in HFmrEF patients should help to identify high-risk subgroups and adjust treatment strategies.

### 3.3 Application of Left Atrial Strain in Heart Failure With Reduced Ejection Fraction

In HFrEF, the LA bears an additional burden under chronic high filling pressure conditions. Varying degrees of elevated LA pressure and dilation are almost universal in HFrEF patients, often resulting in significantly reduced LAS [28]. LAS, particularly left atrial reservoir strain rate (LASRr), has become an important parameter for evaluating HFrEF patients. Studies demonstrate that LASRr correlates closely with both LA and LV filling pressures, serving as a useful surrogate marker for the estimation of LV diastolic function. This is particularly valuable in cases of AF or significant valvular disease, where conventional E/A wave parameters lose reliability [29].

While atrial dysfunction was previously thought to be more prevalent in HFpEF, recent studies suggest that HFrEF patients may exhibit more severe “atrial cardiomyopathy”. For instance, a systematic review and meta-analysis by Jin et al. pooled data across studies and showed that LAS parameters—particularly LASRr—were significantly lower in HFrEF than in HFpEF, indicating more severe intrinsic LA dysfunction in systolic HF [28]. Additionally, common mitral regurgitation (MR) in HFrEF (functional MR) exacerbates LA volume overload and further reduces LASr.

LAS holds unique prognostic value in HFrEF, with multiple studies showing that LAS levels in HFrEF patients predict adverse outcomes from HF. For instance, Park et al. [30] showed that LAS at admission in patients with acute decompensated HFrEF was a significant predictor of HF rehospitalization and mortality post-discharge. Similarly, a cohort study by Bouwmeester et al. [31] found that HFrEF patients with reduced left atrial reservoir strain had poorer cardiac function classification and a significantly increased risk of cardiovascular events during long-term follow-up.

It should be noted that arrhythmias such as AF frequently coexist in HFrEF patients, thereby complicating the measurement of LAS. Furthermore, given the predominantly severe LA dysfunction in this population, LAS may exhibit a “floor effect”, whereby many patients have very low values. Thus, careful selection of sensitive indicators (such as strain rate) is necessary to differentiate risk strata within this cohort [32]. Overall, LAS in HFrEF validates the concept that “the atrium is also a key contributor to HF” [28]. In the future, standardized LAS measurement may be integrated into the management of HFrEF to identify high-risk patients, guide interventions (such as AF and thrombosis prevention), and monitor treatment response.

### 3.4 Critical Appraisal and Evidence Gaps in Heart Failure

Although accumulating evidence supports the diagnostic and prognostic relevance of LAS across the HF spectrum, several limitations remain. Most studies are observational and single-center, with heterogeneous populations and imaging protocols. Reported LAS cut-off values vary substantially (approximately 20–25% in HFpEF), reflecting inter-vendor variability and differences in acquisition methodology. Prospective validation of the incremental value of LAS beyond established markers such as natriuretic peptides and global longitudinal strain (GLS) is still limited. In HFrEF, advanced atrial remodeling and frequent atrial fibrillation may attenuate risk discrimination due to potential floor effects. Standardized measurement protocols and large multicenter outcome-driven studies are required to define robust reference ranges and clinically actionable thresholds.

## 4. Application of Left Atrial Strain in Atrial Fibrillation

AF is the most common sustained arrhythmia in clinical practice, significantly increasing the risk of stroke, HF, and all-cause mortality. AF directly impairs LA function, with rapid, irregular AF waves causing the LA to lose effective contraction. Over time, this leads to structural remodeling (dilatation, fibrosis) and electrical remodeling, causing “AF cardiomyopathy”. LAS provides a quantitative assessment of this AF-related atrial dysfunction, offering potential value for risk stratification and management of AF [33].

Atrial cardiomyopathy has emerged as a unifying concept describing atrial structural, functional, and electrical remodeling that predisposes to AF initiation, arrhythmia burden, adverse outcomes, and variable response to therapy. The recent multinational EHRA/HRS/APHRS/LAHRS consensus statement revisited and refined this framework, emphasizing that AF often represents a manifestation of an underlying atrial disease process rather than an isolated rhythm disorder [34]. In this context, LAS—particularly LA reservoir strain (LASr)—may serve as a feasible, noninvasive marker of atrial mechanical dysfunction and remodeling. A secondary analysis of EAST-AFNET 4 further reported a high prevalence and graded severity of atrial cardiomyopathy in patients with recently diagnosed AF and stroke risk factors, and demonstrated its association with early rhythm-control strategies, supporting the clinical relevance of substrate characterization beyond rhythm status alone [35].

Reduced LAS is closely associated with the onset and progression of AF. During AF episodes, conventional echocardiography cannot detect parameters such as the A wave due to the absence of atrial mechanical activity. However, LAS can still assess residual function through methods such as the averaging of multiple cardiac cycles [14]. Studies have shown that LAS is significantly lower in AF patients than in individuals in sinus rhythm, and that it correlates with increased LA pressure and the degree of atrial fibrosis [36]. The reduction in LAS is considered to reflect impaired LA compliance. Reduced LAS is considered indicative of underlying atrial myocardial injury and serves as a predictive marker for the onset and persistence of AF. For instance, in a prospective study of the general population, individuals with decreased baseline LAS exhibited a significantly elevated risk of first-time AF onset. A systematic review published in JACC: Cardiovascular Imaging synthesized the results from multiple studies and concluded that longitudinal LAS independently predicts the risk of new-onset AF across diverse populations [36,37]. This includes individuals undergoing routine physical examinations, post-stroke patients, post-myocardial infarction patients, and post-cardiac surgery patients. Hauser et al. [38] found that LAS independently predicts the onset of AF, even in patients without LA enlargement and with normal LV systolic function.

LAS can also predict the recurrence risk following AF catheter ablation or cardioversion. Significant pre-ablation LAS reduction often indicates extensive matrix remodeling, which increases the likelihood of post-ablation recurrence. A 2024 study by Zeng et al. [39] demonstrated that patients with significantly reduced preoperative LAS (particularly systolic strain) undergoing radiofrequency ablation for paroxysmal AF exhibited markedly higher AF recurrence rates at 6–12 months postoperatively. Similarly, a cohort study published in Europace reported that LASr is a significant predictor of early AF recurrence following electrical cardioversion [40]. Therefore, LAS can help to identify patients with severely diseased atrial myocardium and potentially limited ablation success rates during decision-making for invasive AF treatment, thereby optimizing patient selection and perioperative management.

LAS correlates with AF thromboembolic risk. Risk assessment for LA appendage thrombus in AF patients currently relies primarily on clinical scores such as CHA_2_DS_2_-VASc. However, some authors have suggested that decreased LAS may indicate intra-atrial blood stasis and a propensity for thrombosis. Abdelhamid et al. [41] reported that LAS analysis provides additional predictive value for asymptomatic LA appendage thrombus in non-valvular AF patients. Although LAS has not yet been incorporated into anticoagulation decision-making for AF, this imaging metric holds promise for more precise assessment of thromboembolic risk in AF in the future.

Beyond chronic remodeling, LAS may capture transient mechanical dysfunction following cardioversion (atrial stunning) [42]. Reduced reservoir strain after restoration of sinus rhythm reflects delayed mechanical recovery and may contribute to early thromboembolic risk. Emerging data suggest that LASr is more strongly associated with left atrial appendage thrombus than conduit strain, indicating phase-specific prognostic implications [43]. Furthermore, reduced pre-procedural LA strain has been shown to predict rhythm outcomes following AF ablation, suggesting LAS may have utility in refining rhythm-control strategies and post-procedure monitoring [44].

Substantial evidence supports the use of LAS for predicting AF onset, assessing the risk of recurrence, and inferring thromboembolic risk and prognosis [45,46,47,48]. However, the application of LAS in AF remains primarily confined to the research stage. It should be noted that many studies of LAS in AF are observational and performed in selected populations, with variability in rhythm status at the time of imaging. Consequently, the optimal timing and methodology for LAS assessment in AF, as well as its role in guiding rhythm-control strategies, remain incompletely defined. In addition to reservoir strain, conduit strain and strain-rate parameters (LASRr, LASRcd, LASRct) may provide complementary mechanistic information, particularly in arrhythmic settings where loading conditions fluctuate. Strain rate may better reflect intrinsic atrial myocardial properties. However, current evidence remains limited and less standardized, and most clinical outcome data have therefore focused on reservoir strain.

## 5. Application of Left Atrial Strain in Hypertension

Hypertension can lead to progressive LV diastolic dysfunction and increased LA load, resulting in subclinical cardiac impairment even when the ejection fraction remains normal. LAS can detect early LA functional alterations in hypertensive patients with high sensitivity [32,49,50]. Even with normal LA dimensions, studies indicate that LASr is markedly lower in hypertensive patients compared to healthy controls, with a more pronounced decrease in those with concomitant diabetes [51]. Mondillo et al. [51] compared LAS in patients with isolated hypertension, diabetes, and healthy individuals. These authors found that LASr in the hypertensive group was significantly lower than in the control group (~29% vs. ~40%, respectively), suggesting that impaired LA function precedes structural remodeling [51]. A subsequent 2022 study by Taamallah et al. [52] further confirmed that hypertensive patients, even without LA enlargement, exhibit reduced reservoir and conduit strain compared to healthy individuals, preceding abnormalities in conventional echocardiographic parameters.

Beyond diagnostic utility, LAS can assess the severity of hypertension-related cardiac damage and treatment efficacy. A study of 208 hypertensive patients found that even in those without LV hypertrophy, LASr and conduit strain were significantly lower than in healthy controls (approximately 30% vs. 35%, and 15% vs. 20%, *p* < 0.05), while LASct showed no significant difference [53]. Abnormal circadian rhythms in hypertension also affect LA function: A 2022 study of 290 hypertensive patients indicated that nocturnal non-dipper patients exhibited significantly higher LA stiffness indices than dipper patients, with markedly reduced values across all phases of LAS (LASr, LAScd, and LASct) [54].

Blood pressure control helps to improve LAS parameters. In a 2024 prospective study, Mousa et al. [55] reported that newly diagnosed hypertensive patients undergoing antihypertensive therapy showed significant improvements in LASr, LASct, E/e’ ratio, and LA stiffness index during follow-up compared to pre-treatment levels. This suggests that effective blood pressure control may partially reverse adverse changes in LA function. Additionally, a small-sample study of patients with resistant hypertension demonstrated that adding spironolactone diuretic therapy for 6 months significantly increased LA systolic strain (from 16.3% to 17.8%, *p* < 0.05), with a trend toward increased LASr [56]. This outcome was partially independent of aldosterone levels, suggesting that renin–angiotensin–aldosterone system modulation may improve LA function by reducing myocardial fibrosis. In summary, LAS analysis in hypertensive patients can assist with early identification of cardiac target organ damage, guide intensified treatment, and evaluate intervention efficacy, thus providing novel imaging evidence for hypertension prevention and management strategies.

However, most of the evidence to date in hypertensive populations derives from cross-sectional studies, limiting causal inference. Variability in blood pressure control, presence of comorbidities (e.g., diabetes), and differences in antihypertensive therapy may influence reported strain values. Whether early impairment of LAS can be translated into improved risk stratification or earlier intervention in hypertension remains to be determined.

## 6. Application of Left Atrial Strain in Coronary Heart Disease

CHD, encompassing acute coronary syndromes and chronic myocardial ischemia remains the leading cause of cardiovascular mortality worldwide [57]. Accurate assessment of both immediate and long-term risks is crucial for the development of treatment strategies in CHD patients. However, traditional risk assessment primarily relies on indicators such as LV function and infarct size, which may not comprehensively reflect overall cardiac function. As an emerging parameter, LAS has been studied to enhance the evaluation system of CHD patients [58].

LAS metrics have shown predictive value in acute coronary syndrome (ACS) patients. A 2024 study by Pedersson et al. [59] followed ACS patients for long-term survival outcomes, revealing that both LASr and LASct were independent predictors of all-cause mortality. Specifically, a 1% decrease in LASr was associated with an approximately 4% increase in mortality risk (HR 1.04). This correlation persisted even after adjusting for patients with normal LA volume indices, suggesting that subtle variations in LA function can significantly impact ACS prognosis, and LAS provides additional risk information beyond LA size.

LAS can also predict adverse outcomes such as HF following myocardial infarction. A 2024 study by Ricken et al. [60] followed patients with ST-segment elevation myocardial infarction (STEMI) for nearly 9 years. These authors found that acute left atrial reservoir strain rate (LASRr) provided incremental predictive value for long-term HF-related events [60]. Adding LASRr to a baseline predictive model comprising age, sex, blood pressure, and ejection fraction significantly increased the ability to predict cardiovascular death, new-onset HF, and HF-related rehospitalization [60]. Furthermore, studies by Tangen et al. (2024) [61] and Sikora-Frac et al. (2025) [62] in a cohort of 501 acute myocardial infarction (MI) patients (some with concomitant AF) also found that reduced LAS is an adverse prognostic factor for cardiovascular death and HF. These results indicate that LA functional decline portends poor disease prognosis regardless of whether MI patients have concomitant arrhythmias.

LAS also serves as an indicator for assessing diastolic dysfunction caused by ischemia. Patients with coronary artery disease often exhibit impaired LV diastolic function, manifested as elevated filling pressures. Traditional echocardiographic parameters like E/e’ have limitations, whereas LAS offers a novel perspective. A 2024 study by Tu et al. [63] in patients with coronary artery disease found that combining LASR with the E/e’ ratio more accurately predicted elevated LV filling pressures, showing superior correlation with catheter measurements than either parameter alone. This finding suggests that LA function is highly sensitive to pressure load changes in ischemic myocardial lesions, and that combined assessment enhances the diagnostic accuracy for diastolic dysfunction.

Numerous recent studies have demonstrated that reduced LAS significantly correlates with mortality risk in ACS patients and the occurrence of HF after myocardial infarction [64]. However, evidence supporting LAS in CHD is predominantly derived from acute coronary syndrome cohorts, with limited data in stable coronary artery disease. Differences in infarct location, revascularization timing, concomitant AF, and LV remodeling may substantially affect LA strain measurements. Reported prognostic thresholds vary across studies and acquisition platforms. Whether LAS provides incremental prognostic value beyond infarct size, ventricular dysfunction, and established risk models requires further prospective validation.

## 7. Application of Left Atrial Strain in the Assessment of Chemotherapy-Related Cardiac Toxicity

As the efficacy of tumor treatment improves, cardiac toxicity induced by anticancer drugs has attracted increasing attention. Agents such as anthracyclines and trastuzumab can cause myocardial injury, leading to cardiomyopathy and HF. Since reduced LVEF often represents a late manifestation of cardiac toxicity, recent focus has shifted towards monitoring subclinical cardiac function changes during asymptomatic stages in order to enable timely intervention. As a sensitive indicator of myocardial diastolic function, LAS has unique advantages in the early detection of cancer treatment-related cardiac toxicity [65].

GLS of the LV can detect subclinical systolic dysfunction even when LVEF remains normal. Consequently, GLS has been progressively adopted for cardiac monitoring in cancer patients [66]. However, GLS primarily reflects systolic function and cannot directly assess diastolic function. Adverse changes in myocardial diastolic function often signal cardiac toxicity at an earlier stage [67]. LAS can bridge this gap by acting as a barometer of LV filling pressure and diastolic function.

Several prospective studies demonstrate that in patients receiving highly cardiotoxic chemotherapy such as anthracyclines or HER2 inhibitors, significant decreases in left atrial strain occur after only a few treatment cycles, even when LVEF and LV GLS remain unchanged [68,69]. For example, the mean decline in LASr in breast cancer patients receiving anthracycline-based chemotherapy can reach several percentage points after a few cycles, preceding alterations in GLS and LVEF [69].

Baseline LAS levels may also help to identify high-risk patients. Low LAS prior to chemotherapy initiation suggests impaired cardiac reserve function, increasing the likelihood of subsequent cardiac events during treatment. Several studies have also shown that patients whose LAS fails to recover after treatment completion exhibit a higher risk of developing HF or persistent cardiac dysfunction during follow-up, necessitating long-term monitoring and intervention [70]. Therefore, LAS can be used not only for early detection of cardiotoxicity, but also for post-treatment risk assessment.

While early changes in LAS appear promising for the detection of subclinical cardiotoxicity, current evidence is derived from relatively small, single-center studies. Differences in treatment regimens, cumulative drug exposure, and timing of strain assessment may substantially influence reported findings. In addition, inter-vendor variability in strain acquisition limits comparability of LAS thresholds. Conflicting data regarding the temporal sequence of LAS, GLS, and LVEF changes further highlight the need for standardized surveillance protocols.

## 8. Application of Left Atrial Strain in Valvular Heart Disease

Valvular heart disease (VHD) can cause pressure or volume overload that directly affects the LA. Chronic valvular disease (such as mitral and aortic valve lesions) frequently leads to LA enlargement and fibrosis. Changes in LA function not only indicate disease severity but also correlate closely with patient prognosis. In recent years, LAS has been employed to assess the impact of valvular disease on LA function, aiding clinical decision-making [71].

### 8.1 Mitral Regurgitation

In chronic mitral regurgitation (MR), the LA is subjected to prolonged volume overload, leading to marked LA enlargement and impaired function. Studies indicate that LASr serves as a crucial parameter for assessing prognosis and determining the optimal timing for surgery in patients with chronic severe MR. Debonnaire et al. [72] conducted a follow-up analysis of 121 patients with severe MR and reported that LASr is an independent predictor of reaching surgical criteria: a 1% decrease in LASr indicated an increased likelihood of meeting surgical criteria, and patients with LASr ≤24% showed a significantly higher risk of poor prognosis or symptoms development during follow-up. Notably, patients with preoperative LASr ≤24% demonstrated markedly worse long-term postoperative survival rates compared to those with higher LASr, even after timely valve surgery [72]. Thus, the incorporation of LASr into existing surgical criteria (e.g., symptoms, AF status, LV function) increases the accuracy of determining the optimal timing for surgery [73].

LAS also helps to identify MR patients who are prone to postoperative recurrence or limited benefit. van Garsse et al. [74] reported that ischemic MR patients with lower preoperative LAS were more susceptible to MR recurrence after valvuloplasty (annuloplasty). Specifically, patients with lower peak LAS and strain rate exhibited higher rates of moderate-to-severe MR recurrence within the postoperative period [75]. Work by Lisi et al. [76] suggests that patients with severely impaired LA function may have irreversible atrial-ventricular remodeling, where valve repair alone yields limited efficacy, thereby necessitating closer postoperative follow-up and adjunctive therapy [77].

For patients with moderate asymptomatic MR, LAS helps to identify high-risk individuals, thus guiding early intervention. Cameli et al. [73] conducted a prospective study involving 395 patients with primary moderate degenerative MR (asymptomatic). Patients who experienced cardiovascular events (primarily HF and AF) during follow-up were observed to have significantly lower baseline LAS reserve compared to event-free patients [73]. Therefore, early surgical intervention should be considered for asymptomatic patients with moderate MR and markedly reduced LAS in order to prevent irreversible myocardial damage.

### 8.2 Mitral Stenosis

Rheumatic mitral stenosis (MS) leads to increased LA pressure load, making it one of the earliest recognized diseases and highlighting the importance of LA function. LAS quantitatively assesses the degree of impairment in left atrial function caused by MS and reflects disease severity. Studies indicate that patients with severe MS exhibit significantly lower LASr, LAScd, and LASct compared to those with mild MS or healthy controls [78]. These reduced strain values correlate closely with the severity of transvalvular pressure gradient and secondary pulmonary hypertension. Therefore, assessing MS via LAS can not only reveal the extent of LA dysfunction but may also serve as an indirect indicator of stenotic burden and pulmonary circulation involvement.

Clinically, symptomatic MS often indicates the need for interventional (percutaneous balloon valvuloplasty) or surgical treatment. However, many patients remain asymptomatic at rest. LAS holds promise in identifying asymptomatic patients with markedly reduced LA reserve function. Such patients often develop symptoms during exercise stress and exhibit poorer prognosis, warranting serious consideration for intervention. Preliminary studies suggest that patients with markedly reduced LAS may derive greater benefit from intervention, even those with a lower NYHA functional class [78]. In summary, LAS can serve as a complementary assessment tool in the management of MS, refining risk stratification and guiding treatment decisions.

### 8.3 Aortic Valve Stenosis

Aortic stenosis (AS) primarily causes increased LV pressure overload, but its impact on the LA is equally profound. LAS has been demonstrated to hold significant prognostic value in AS patients. Tan et al. [79] reported that peak LASr can independently predict the incidence of major cardiovascular events in patients with severe AS, with superior efficacy to many traditional echocardiographic parameters. The study proposed a set of strain-based risk thresholds (LASr <20%, LAScd <6%, and absolute LASct <12%) wherein patients exhibited a significantly elevated risk of adverse events such as HF and death.

A systematic review encompassing 18 studies and 2660 AS patients further supported this conclusion. A 1% decrease in LASr was found to be associated with a significant increase in the risk of death, heart failure, or the need for surgery [80]. Thus, LAS quantitatively reflects the adverse cardiac impact of AS and can be used for precise patient risk stratification. LAS can also be used to evaluate the efficacy of AS interventions. Transcatheter aortic valve replacement (TAVR) has become one of the primary treatments for severe AS. Interestingly, it has been reported that LAS often improves significantly within months after relieving aortic valve pressure overload, even preceding LA volume reduction [81]. This suggests that LA function can undergo reversible changes in the short term, and LAS can serve as a sensitive indicator of the reversal of cardiac remodeling [82]. LAS may provide incremental value in discordant aortic stenosis, particularly in low-flow, low-gradient cases where conventional measures yield conflicting estimates of severity. Reduced LAS likely reflects chronic pressure overload and advanced atrial remodeling, which may aid risk stratification in this subgroup.

### 8.4 Heterogeneity in Valve Etiology and Hemodynamic Status

LAS impairment in valvular heart disease reflects complex interactions between pressure or volume overload and atrial remodeling. However, heterogeneity in valve etiology (degenerative versus rheumatic), disease stage, and timing of intervention complicates comparison across studies. Reported strain thresholds differ considerably, partly due to inter-vendor variability and inconsistent acquisition protocols. In aortic stenosis, discordant grading and flow status further introduce methodological complexity. Prospective multicenter validation is required before LAS can be incorporated into routine decision algorithms.

## 9. Application of Left Atrial Strain in Cardiomyopathy

### 9.1 Application of Left Atrial Strain in Cardiac Amyloidosis

Cardiac amyloidosis (CA) is a representative restrictive cardiomyopathy. Amyloid fiber deposition in the myocardial interstitium leads to ventricular stiffness and hypertrophy, resulting in early diastolic dysfunction and elevated filling pressures, followed by LA enlargement and impaired function [83,84]. Since amyloid can also deposit in the interatrial septum, patients with CA often exhibit significant atrial cardiomyopathy. LAS is a sensitive indicator of the degree of atrial involvement, making it particularly valuable for diagnosis and differential diagnosis of CA.

On one hand, LAS aids in the early detection of atrial dysfunction caused by CA. In a 2023 study, Monte et al. [84] compared strain measurements among wild-type transthyretin amyloidosis (ATTR) amyloidosis patients, hypertrophic cardiomyopathy (HCM) patients, and healthy controls. Results showed that all three phases of LAS—storage, transmission, and contraction—were significantly lower in the amyloidosis group compared to the HCM group and healthy controls [84]. These strain metrics were markedly impaired, indicating that CA compromises atrial function earlier than it affects LV systolic function [85]. Conversely, in purely pressure-overload hypertrophic diseases like HCM, the reduction in LAS is relatively less severe.

LAS can also contribute to the differential diagnosis of CA. In a 2024 study, Mattig et al. [86] reported that LAS can help differentiate between two hypertrophic cardiomyopathies, CA and Fabry disease: LASr <20% indicates CA with 90% sensitivity and 64% specificity. This relatively straightforward threshold provides clinically useful guidance. Furthermore, a 2022 study by Bandera et al. [87] confirmed via CMR and ultrasound assessment that LA “infiltration” due to amyloid deposition significantly reduces LAS, with the degree of reduction correlating with the patients’ HF symptoms and survival rates. Therefore, LAS not only serves as a diagnostic indicator for CA but also reflects disease severity and holds prognostic significance. A retrospective cohort study by Oike et al. [88] found that preserved LASr was a clinically significant predictor of lower cardiovascular mortality in this cohort.

### 9.2 Application of Left Atrial Strain in Hypertrophic Cardiomyopathy

HCM is a common hereditary heart disease caused primarily by mutations in cardiac myosin genes and leading to myocardial hypertrophy and fibrosis. HCM typically presents with preserved or hyperdynamic LV systolic function, but is accompanied by impaired diastolic function, elevated filling pressures, and mitral valve abnormalities. All of these factors contribute to sustained LA pressure overload [89,90]. LA enlargement is common in HCM patients and is closely associated with adverse outcomes such as AF and stroke.

Beyond LA size, LAS can be used for the early detection of atrial dysfunction in HCM patients [91]. Hinojar et al. [92] demonstrated that reduced LAS, particularly diminished LASr function assessed via feature tracking, holds significant prognostic value in HCM patients. Those with more severe LA dysfunction experienced higher rates of adverse cardiac events during follow-up [92]. Tayal et al. [93] further discovered that LAS in HCM patients correlates closely with the extent of myocardial fibrosis and diastolic dysfunction. Patients exhibiting extensive LV hypertrophy and fibrosis typically present with lower LAS [93]. Consequently, LAS serves as a comprehensive marker of disease severity in HCM, reflecting both structural alterations in the LA and hemodynamic burden.

Phenotype-specific differences may also influence LAS patterns in HCM. Patients with obstructive HCM (HOCM) have been reported to exhibit more pronounced impairment of left atrial strain compared with non-obstructive forms, likely reflecting higher LV outflow tract gradients and greater diastolic burden [94]. In this context, reduced reservoir strain appears to parallel the severity of hemodynamic compromise and atrial remodeling. Conversely, in non-obstructive HCM, LAS impairment may be more closely related to the extent of myocardial fibrosis and chronic structural remodeling rather than dynamic obstruction. These findings suggest that LAS may capture distinct pathophysiological mechanisms across HCM phenotypes and contribute to refined risk stratification.

### 9.3 Application of Left Atrial Strain in Dilated Cardiomyopathy

Dilated cardiomyopathy (DCM) is a cardiomyopathy characterized by LV enlargement, impaired LV systolic function, and cardiac remodeling. Its etiology can be categorized into ischemic and non-ischemic types [95]. Traditionally, LA assessment in DCM patients has primarily focused on LA volume, LA function, and AF risk. A 2022 study of 497 patients found that CMR-derived LAS was an independent predictor of all-cause mortality or transplantation. For instance, a 1% increase in LA reservoir strain was associated with an approximately 5% reduction in the risk of death or transplantation (hazard ratio [HR] ~0.95 per 1% increase) [96]. LAS added prognostic value even after accounting for other factors such as age, ejection fraction (EF), and myocardial scarring [96]. Similarly, in a 2024 multicenter study on 811 patients with ischemic or non-ischemic DCM, Fong et al. [97] reported that lower LAS correlated with higher mortality or HF hospitalization rates, thus establishing LAS as a significant independent prognostic marker in DCM patients.

### 9.4 Phenotype-Specific Variability and Imaging Considerations

Studies assessing LAS in cardiomyopathies are frequently derived from phenotype-specific cohorts with relatively limited sample sizes, introducing potential selection bias. Substantial heterogeneity exists between obstructive and non-obstructive HCM, as well as between ischemic and non-ischemic DCM, with varying degrees of myocardial fibrosis influencing atrial mechanics. Moreover, differences in imaging modality (echocardiography versus CMR feature tracking) and inter-vendor software algorithms limit direct comparability of reported strain values. Whether LAS provides incremental prognostic information beyond established ventricular markers across diverse cardiomyopathy phenotypes remains incompletely defined.

## 10. Clinical Integration of Left Atrial Strain

From a clinical perspective, LAS may serve as a complementary imaging biomarker to refine risk stratification across multiple cardiovascular conditions. In HF, particularly HFpEF, LAS may assist in phenotyping atrial cardiomyopathy and identifying patients with advanced diastolic dysfunction despite preserved LVEF. In AF, impaired LAS has potential utility in predicting rhythm-control outcomes and recurrence after catheter ablation. In valvular heart disease, LAS may provide incremental information to support optimal timing of intervention before the occurrence of irreversible atrial remodeling. Furthermore, early changes in LAS may facilitate the detection of subclinical myocardial injury in patients undergoing cardiotoxic cancer therapy. Although these applications require further investigation, they illustrate potential pathways by which LAS could be integrated into routine echocardiographic assessment as an adjunct to conventional parameters. Table 2 (Ref. [19,20,38,39,51,52,58,59,65,72,79,84,88]) summarizes representative clinical studies that have evaluated the application of LAS across different cardiovascular diseases. To facilitate clinical interpretation, a practical summary of potential LAS applications across major cardiovascular conditions is provided in Table 3.

**Table 2.** **Representative clinical studies evaluating LAS in cardiovascular diseases**.

First author	Year	Study design	Disease category	LAS parameter(s)	Primary endpoint/key outcome	Main findings
Ye et al. [19]	2020	Prospective cohort	HFpEF	LASr	HFpEF vs. controls differentiation	LASr distinguished HFpEF from controls and correlated with LV diastolic dysfunction.
Carluccio et al. [20]	2023	Machine-learning multicenter cohort	Heart failure (mixed)	LASr	Composite HF outcomes	LAS-based clustering improved phenotyping and risk stratification compared with conventional parameters.
Hauser et al. [38]	2022	Prospective community cohort	Atrial fibrillation	LASr	Incident AF	LASr independently predicted incident AF despite normal LA size and preserved LV function.
Zeng et al. [39]	2024	Prospective study	Atrial fibrillation	LASr, LASct	Post-ablation AF recurrence	Reduced pre-procedural LAS predicted AF recurrence after catheter ablation.
Mondillo et al. [51]	2011	Cross-sectional	Hypertension & diabetes	LASr	Subclinical LA dysfunction	LASr detected early LA functional impairment before structural enlargement.
Taamallah et al. [52]	2022	Prospective cohort	Hypertension	LASr, LAScd	Early functional impairment	LAS abnormalities preceded changes in conventional diastolic parameters.
Li et al. [58]	2022	Retrospective study	Acute coronary syndrome	LASr	MACE	Reduced LASr independently predicted major adverse cardiovascular events after ACS.
Pedersson et al. [59]	2024	Retrospective study	Coronary artery disease	LASr, LASct	All-cause mortality	LAS independently predicted long-term all-cause mortality, irrespective of LV function.
Park et al. [65]	2020	Retrospective study	Cardiotoxicity	LASr	Subclinical CTRCD detection	Early decline in LASr identified chemotherapy-related cardiotoxicity before GLS deterioration.
Oike et al. [88]	2021	Retrospective cohort	ATTR amyloidosis	LASr	Cardiovascular mortality	Preserved LASr predicted lower CV mortality; LVEF and E/e’ were not predictive.
Monte et al. [84]	2023	Multicenter cohort	CA vs. HCM	LASr, LAScd, LASct	Disease differentiation	LAS parameters effectively distinguished cardiac amyloidosis from hypertrophic cardiomyopathy.
Debonnaire et al. [72]	2013	Prospective surgical cohort	Mitral regurgitation	LASr	Surgical timing & postoperative outcome	LASr predicted optimal timing for surgery and long-term postoperative prognosis.
Tan et al. [79]	2023	Prospective cohort	Aortic stenosis	LASr	MACE	LASr independently predicted adverse cardiovascular events in patients with severe aortic stenosis.

CVD, cardiovascular disease; ACS, acute coronary syndrome; HCM, hypertrophic cardiomyopathy; ATTR, transthyretin amyloidosis; MACE, major adverse cardiovascular events; CTRCD, cancer therapy-related cardiac dysfunction; HFpEF, heart failure with preserved ejection fraction; HFrEF, heart failure with reduced ejection fraction; AF, atrial fibrillation; CA, cardiac amyloidosis; LV, left ventricle; LVEF, left ventricular ejection fraction; E/e’, E wave/e' wave.

**Table 3.** **Potential clinical applications of left atrial strain across cardiovascular conditions**.

Clinical condition	LAS application	Evidence level	Key limitations
HFpEF	LASr for diastolic assessment and risk stratification	Moderate	Vendor variability; cut-off inconsistency
HFmrEF/HFrEF	LASr ± LASRr for prognostic assessment	Moderate	Floor effect; rhythm variability
Atrial Fibrillation	LASr for recurrence and thromboembolic risk estimation	Moderate	Rhythm timing; loading dependence
Hypertension	LASr for early detection of atrial dysfunction	Emerging	Predominantly cross-sectional data
Coronary Heart Disease	LASr for post-MI risk stratification	Emerging–Moderate	Cohort heterogeneity
Valvular Heart Disease	LASr for prognostic stratification	Moderate	Flow-dependent variability
Cardiomyopathies	LASr for assessment of atrial involvement	Emerging–Moderate	Small disease-specific cohorts
Cardiotoxicity	LASr for early functional impairment detection	Emerging	Lack of standardized surveillance

## 11. Limitations and Future Prospects

Despite its growing use as a quantitative index of atrial function, the clinical application of LAS is still constrained by several practical issues. The most relevant limitation is the lack of true standardization. Current LAS measurements rely on vendor-specific speckle-tracking algorithms, and differences in tracking logic and post-processing may lead to systematic discrepancies across platforms. Such inter-vendor variability contributes to differences in reported reference ranges and may partly explain the heterogeneity of cut-off values across studies. This lack of harmonization limits direct comparison of prognostic thresholds. Therefore, values reported in individual studies should be interpreted within their specific technical context rather than viewed as universally interchangeable. A second challenge relates to measurement robustness in real-world settings. Although LAS shows good reproducibility under optimal imaging conditions, its reliability remains dependent on image quality and rhythm regularity. In patients with AF or frequent ectopic beats, cycle-to-cycle variability can substantially influence strain values, making interpretation less straightforward. While the averaging of multiple cardiac cycles is commonly adopted, consensus protocols for arrhythmic patients are still lacking.

Beyond these technical considerations, clinically actionable thresholds for LAS remain incompletely defined. Normal reference ranges vary with age and sex, and disease-specific cut-off values differ across cardiovascular conditions and study populations. Consequently, LAS is currently better suited for risk stratification and comparative assessment than for rigid diagnostic decision-making. Finally, the integration of LAS into contemporary guidelines remains limited, as its incremental clinical utility beyond established parameters has not been fully validated in prospective outcome-driven trials. In addition, the cost-effectiveness of routine LAS implementation, including software requirements, acquisition time, and training burden, has not been systematically evaluated. These practical considerations represent important barriers to widespread adoption.

Future efforts should focus on obtaining large-sample data through multicenter collaborations to establish standardized protocols. Large-scale prospective studies are needed to further define reference thresholds and benefit thresholds for LAS across different populations and clinical scenarios, thereby facilitating its integration into routine clinical practice. Future developments, including artificial intelligence assisted strain analysis, vendor-independent software solutions, and integration with multimodality imaging, may further enhance the feasibility and clinical applicability of LAS. Recent studies have demonstrated the feasibility of automated AI-based LAS extraction from echocardiographic datasets, showing promising reproducibility with reduced operator dependency [98,99,100].

## 12. Conclusions

LAS is a noninvasive, quantitative functional indicator that holds great promise in the early diagnosis and risk assessment of cardiovascular diseases. It can predict adverse cardiovascular events, aid in risk stratification, and detect atrial dysfunction at an early stage across various clinical scenarios, including hypertension, HF, AF, cardiomyopathy, and valvular disease. However, its standardized application still requires further validation through large-scale prospective studies.
